# Genome-Wide Specific Selection in Three Domestic Sheep Breeds

**DOI:** 10.1371/journal.pone.0128688

**Published:** 2015-06-17

**Authors:** Huihua Wang, Li Zhang, Jiaxve Cao, Mingming Wu, Xiaomeng Ma, Zhen Liu, Ruizao Liu, Fuping Zhao, Caihong Wei, Lixin Du

**Affiliations:** Institute of Animal Sciences, Chinese Academy of Agricultural Sciences, Beijing, People’s Republic of China, Beijing, China; China Agricultrual University, CHINA

## Abstract

**Background:**

Commercial sheep raised for mutton grow faster than traditional Chinese sheep breeds. Here, we aimed to evaluate genetic selection among three different types of sheep breed: two well-known commercial mutton breeds and one indigenous Chinese breed.

**Results:**

We first combined locus-specific branch lengths and *d_i_* statistical methods to detect candidate regions targeted by selection in the three different populations. The results showed that the genetic distances reached at least medium divergence for each pairwise combination. We found these two methods were highly correlated, and identified many growth-related candidate genes undergoing artificial selection. For production traits, *APOBR* and *FTO* are associated with body mass index. For meat traits, *ALDOA*, *STK32B* and *FAM190A* are related to marbling. For reproduction traits, *CCNB2* and *SLC8A3* affect oocyte development. We also found two well-known genes, *GHR* (which affects meat production and quality) and *EDAR* (associated with hair thickness) were associated with German mutton merino sheep. Furthermore, four genes (*POL*, *RPL7*, *MSL1* and *SHISA9*) were associated with pre-weaning gain in our previous genome-wide association study.

**Conclusions:**

Our results indicated that combine locus-specific branch lengths and *d_i_* statistical approaches can reduce the searching ranges for specific selection. And we got many credible candidate genes which not only confirm the results of previous reports, but also provide a suite of novel candidate genes in defined breeds to guide hybridization breeding.

## Introduction

China is the largest mutton producer in the world. According to 2012 statistics from the Food and Agriculture Organization of the United Nations, China accounts for almost one third of the world's yield of mutton (http://faostat.fao.org/). One reason for this is that there are a large number of Muslim and Mongolian residents in China and mutton is their main meat source. Meanwhile, more and more people of Han Chinese like eating mutton. As the status of mutton increases, so the deficit in the domestic supply of mutton also increases and the annual amount imported becomes ever larger. China does not have its own commercial mutton sheep breed, and the average meat production capacity of traditional Chinese breeds is lower compared with other countries. Therefore, development of a special Chinese sheep breed for meat production is needed.

Meat production traits have significant economic importance. Hybridization can quickly improve the meat quality of Chinese sheep, but cannot stabilize the inheritance of desirable traits. Identification of genomic regions that influence meat performance would enable improvement of local Chinese varieties by cross-breeding. This would have very real significance, not only to improve the weakness in Chinese mutton production, but also to improve to mutton production throughout the world.

To mine for genome selection information, the selection signal method has become popular. For the specific selection of genomic regions, pairwise F_ST_, combined with a haplotype approach, such as REHH (Relative extended haplotype homozygosity), XPEHH (Cross population extended haplotype homozygosity)[1] or RSB (Across pairs of populations)[2] can determine the selection from a population when dealing with two groups. But it is relatively complex for multi-groups. Global F_ST_, applied to multiple groups, cannot determine which breeds have undergone selection. At present, there are two better methods, locus-specific branch lengths (LSBL) and *d*_*i*_, which detect the locus specific divergence for each breed. LSBL is generally suitable for three or four groups [3], whereas *d*_*i*_ is suitable for three or more groups [4].

In our previous study, we identified candidate genes associated with growth and meat production traits by using Illumina Ovine SNP50 BeadChip technology and genome-wide association study (GWAS) methodology to analyze three sheep populations including one indigenous Chinese sheep breed and two well-known commercial mutton sheep breeds [5]. Here we also applied these data to identify artificial selection regions using LSBL and *d*_*i*_ statistics.

## Materials and Methods

### Population samples and quality control

We analyzed SNP (Single-nucleotide polymorphism) data from our previous GWAS [5]. A total of 322 sheep from three breeds, including 61 Chinese Mongolian fat-tailed (CMF), 161 German Mutton Merino (GMM) and 100 African white Dorper (AWD) sheep were analyzed. There were not any family structure and half sib family in the selected sheep. Two SNP sets were used. First, SNPs that did not pass the following three criteria were excluded: (1) SNPs with minor allele frequency > 0.01; (2) Hardy–Weinberg Equilibrium *P-*value > 0.000001; (3) SNPs that were located on autosomes. After quality control, there were 322 individuals and 46,752 SNPs in the genetic diversity analysis dataset. The first SNP set was then pruned using the indep-pairwise option, with a non overlapped window size of 25 SNPs, a step of 5 SNPs, and pairwise r^2^ threshold of 0.1, resulting in 10,260 independent SNP markers. The second SNP set was for population analysis.

### Population analyses

Principal component analysis (PCA) was conducted using snpStats in R (http://cran.r-project.org). We constructed two neighbor-joining trees. One of uncorrected p-distances for individuals using SplitsTree software [6] and one of pairwise F_ST_ for populations using R package *ape* [7].

### Statistical analyses

We first calculated pairwise F_ST_ for each locus of first SNP set between breeds using Genepop 4.2.2 software [8]. Neighbor-joining tree breed-specific population differentiation within 300 kb windows across the 26 autosomes was calculated using Locus-specific branch lengths (LSBL) [3] and *d*_*i*_ statistics [4]. As described in Shriver et al. 2004 [3], LSBL (L_GMM_, L_AWD_, L_CMF_) were calculated from single locus pairwise F_ST_ distances, where L_GMM_ = (GMM-AWD F_ST_ + GMM-CMF F_ST_ − AWD-CMF F_ST_)/2, L_AWD_ = (GMM-AWD F_ST_ + AWD-CMF F_ST_ − GMM-CMF F_ST_)/2 and L_CMF_ = (GMM-CMF F_ST_ + AWD-CMF F_ST_ − GMM-AWD F_ST_)/2. Akey et al. [4] first described how to calculate *d*_*i*_ statistics for each SNP; *d*_*i*_ = $\sum_{j\neq i}\frac{F_{ST}^{ij}-E\left[F_{ST}^{ij}\right]}{sd\left[F_{ST}^{ij}\right]}$, where $E\left[F_{ST}^{ij}\right]$ and $sd\left[F_{ST}^{ij}\right]$ denote the expected value and standard deviation of pairwise F_ST_ values between breeds i and j calculated from all SNPs. Only windows with a minimum of three SNPs were considered. For each breed, windows of significance were determined as those with LSBL or *d*_*i*_ values falling into the 99^th^ percentile of the empirical distribution.

### Gene annotation

We used the latest sheep genome release *Ovis_aries*_*v*3.1 (http://www.livestockgenomics.csiro.au/sheep/oar3.1.php) to identify relationships between significant selection windows and ovine genes. Owing to the structural imperfection and incomplete sheep genome sequence (before October, 2012), we also referenced genomic information of other species such as human, cow, mouse and rat.

## Results

### Population stratification

**Fig 1:**
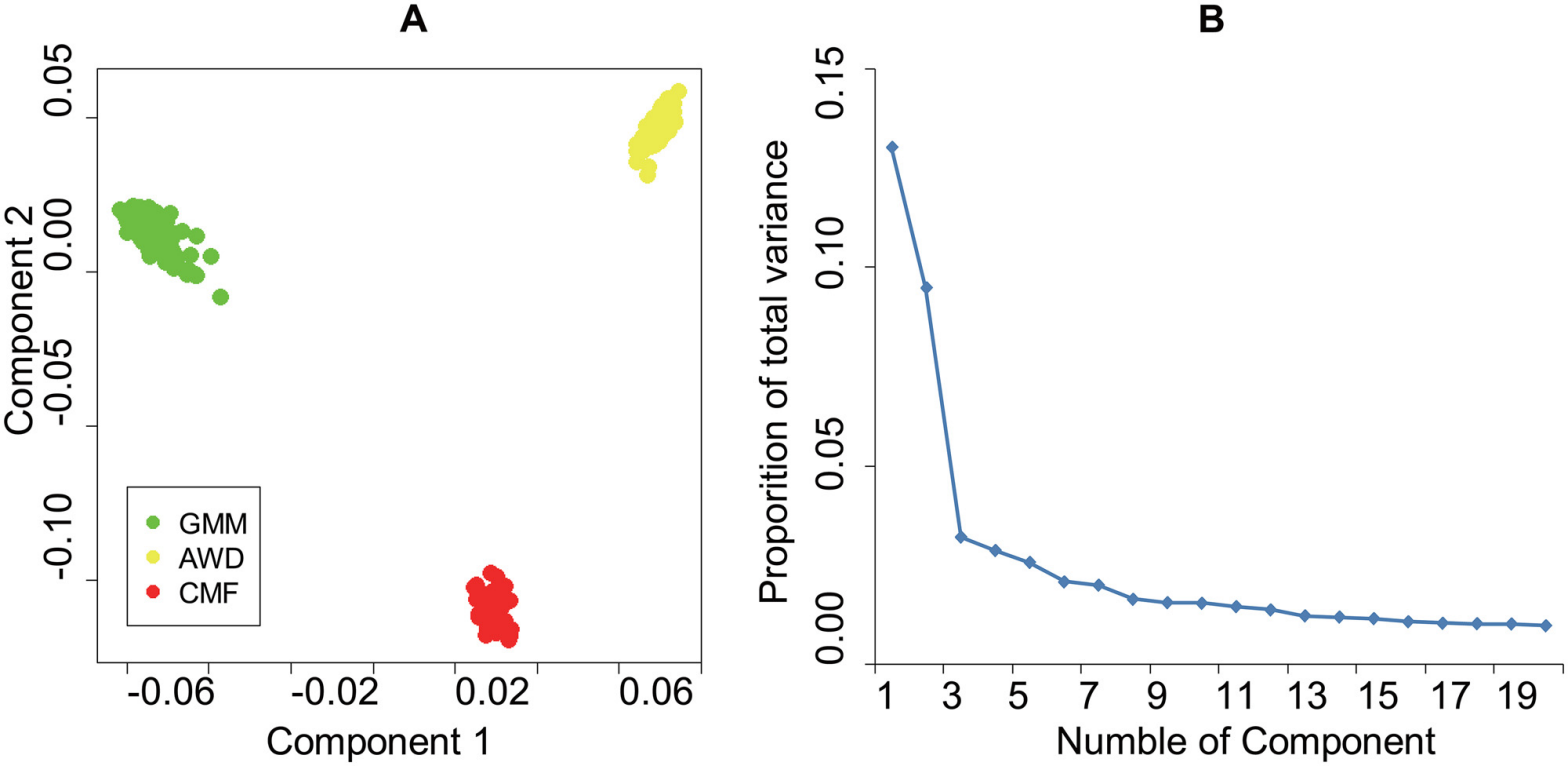
A. Animals clustered on the basis of principal component (PC) analysis using individual genotypes B. Scree-plot of proportion of variance.

In the present study, we first performed principal component analysis on a pruned set of 10,260 genome-wide SNPs, to characterize the pattern of individual clustering in the sample set. As shown in Fig 1, PC1 (which accounts for 13.01% of the total variance) and PC2 (which accounts for 9.47% of the total variance) both separate all three population samples from each other, as the same with the former study[5].

**Fig 2:**
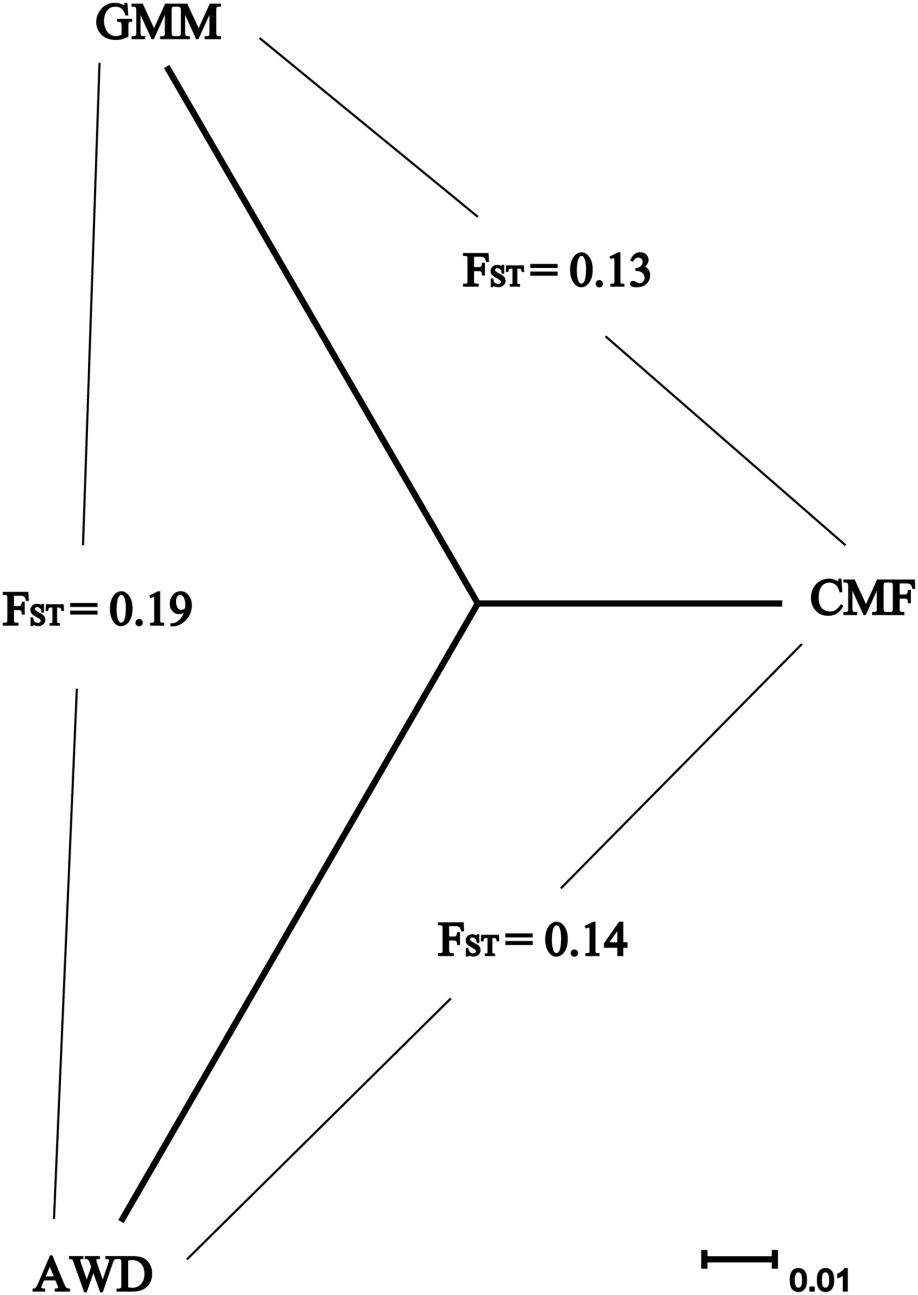
Three-branch phylogeny constructed from pairwise F_ST._

We then calculated pairwise F_ST_ [9] for the SNP data generated from the three sheep population samples (Fig 2). According to Wright’s theory [10], we found medium divergence (F_ST_ = 0.13, F_ST_ = 0.14) between CMF and GMM or AWD populations respectively, and high divergence (F_ST_ = 0.19) between AWD and GMM populations. We constructed a simple three-branch phylogeny from pairwise F_ST_ values (Fig 2) and also a neighbor-joining (NJ) tree among the individuals (S1 Fig). The results clearly showed that there were no conflicts concerning the origins of individuals assigned to each breed.

### Correlation of two locus specific analysis approaches

**Fig 3:**
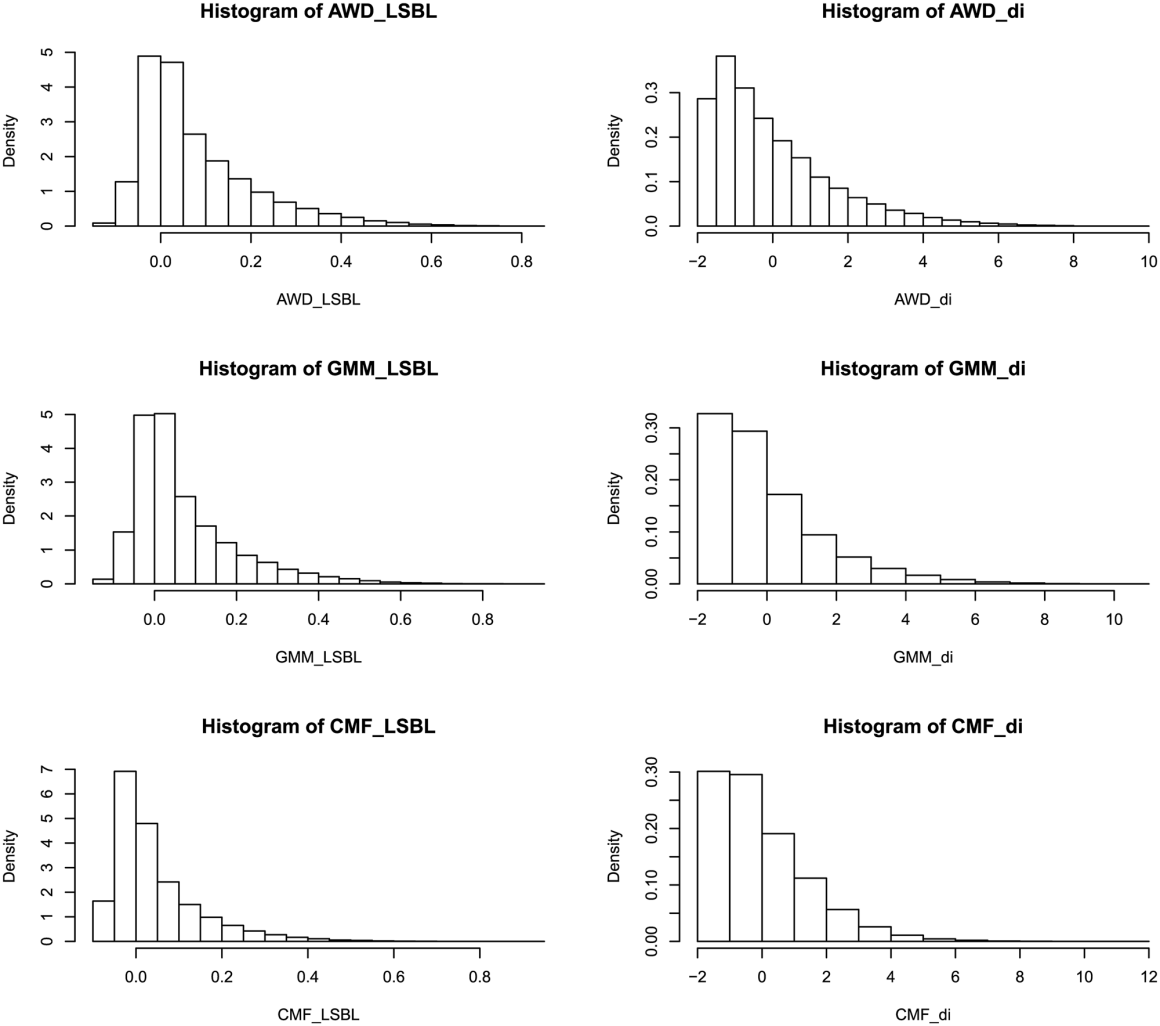
Histogram of the LSBL and *d*_*i*_ statistics distribution for each breed.

Locus-specific branch lengths (LSBL) [3] and *d*_*i*_ statistics [4] are both summary statistical methods to measure the locus specific divergence in allele frequencies for each breed based on unbiased estimates of pairwise F_ST_ [11]. LSBL is suited to the analysis of three populations, and *d*_*i*_ is preferred for analysis of more than three populations. When the populations number is three, both approaches can be used. In this study, we calculated genome-wide LSBL and *d*_*i*_ values. The maximal L_CMF_ and d_CMF_ values were higher than those of the other two breeds (Table 1). Obviously, the mean L_AWD_ and L_GMM_ were higher than L_CMF_, and branch lengths of AWD and GMM were longer than those of CMF (Table 1, Fig 2). In other words, the CMF breed shows more loci having shorter LSBL compared with the other two breeds. Histograms of the distribution of LSBL and *d*_*i*_ statistics for each breed are shown in Fig 3. AWD and GMM have similar LSBL distributions. But GMM and CMF are similar *d*_*i*_ statistics distributions. Further, we used Pearson’s product-moment correlation to estimate the correlation between LSBL and *d*_*i*_ statistics within each breed. All three breeds showed significant correlation (P-value<2.2e-16) between the two approaches. The correlations for AWD (r = 0.85) and GMM (r = 0.84) were higher than that for CMF (r = 0.68).

**Table 1:** The descried of LSBL and *d*_*i*_ values for each breed

Value	Mean(SD)	Min	Max
L_AWD_	0.079(0.13)	-0.113	0.838
L_GMM_	0.071(0.13)	-0.125	0.914
L_CMF_	0.045(0.10)	-0.083	0.940
d_AWD_	-0.003(1.62)	-1.859	9.728
d_GMM_	-0.012(1.61)	-1.809	10.1
d_CMF_	-0.010(1.46)	-1.785	11.352

**Fig 4:**
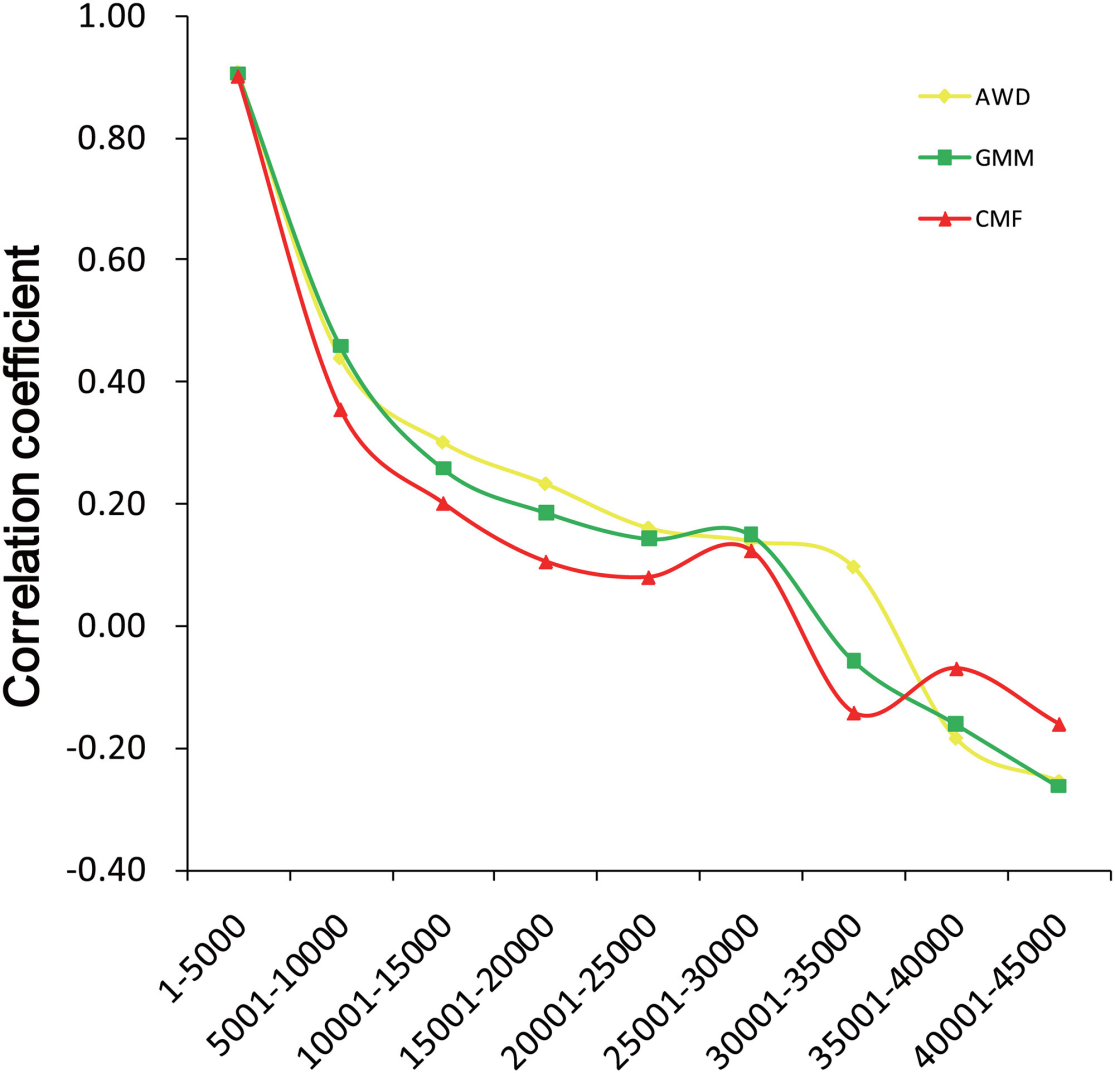
Correlation between LSBL and *d*_*i*_ statistics.

We also investigated the correlation between LSBL and *d*_*i*_ statistics in 5000 SNPs in bin order, from high to low of LSBL value (Fig 4). The highest correlation (r>0.9) occurred in the region of the top 1–5000 SNPs in all breeds. The correlation values then sharply declined in the top 5001–10000 SNPs.

### Detecting breed specific selection regions

**Fig 5:**
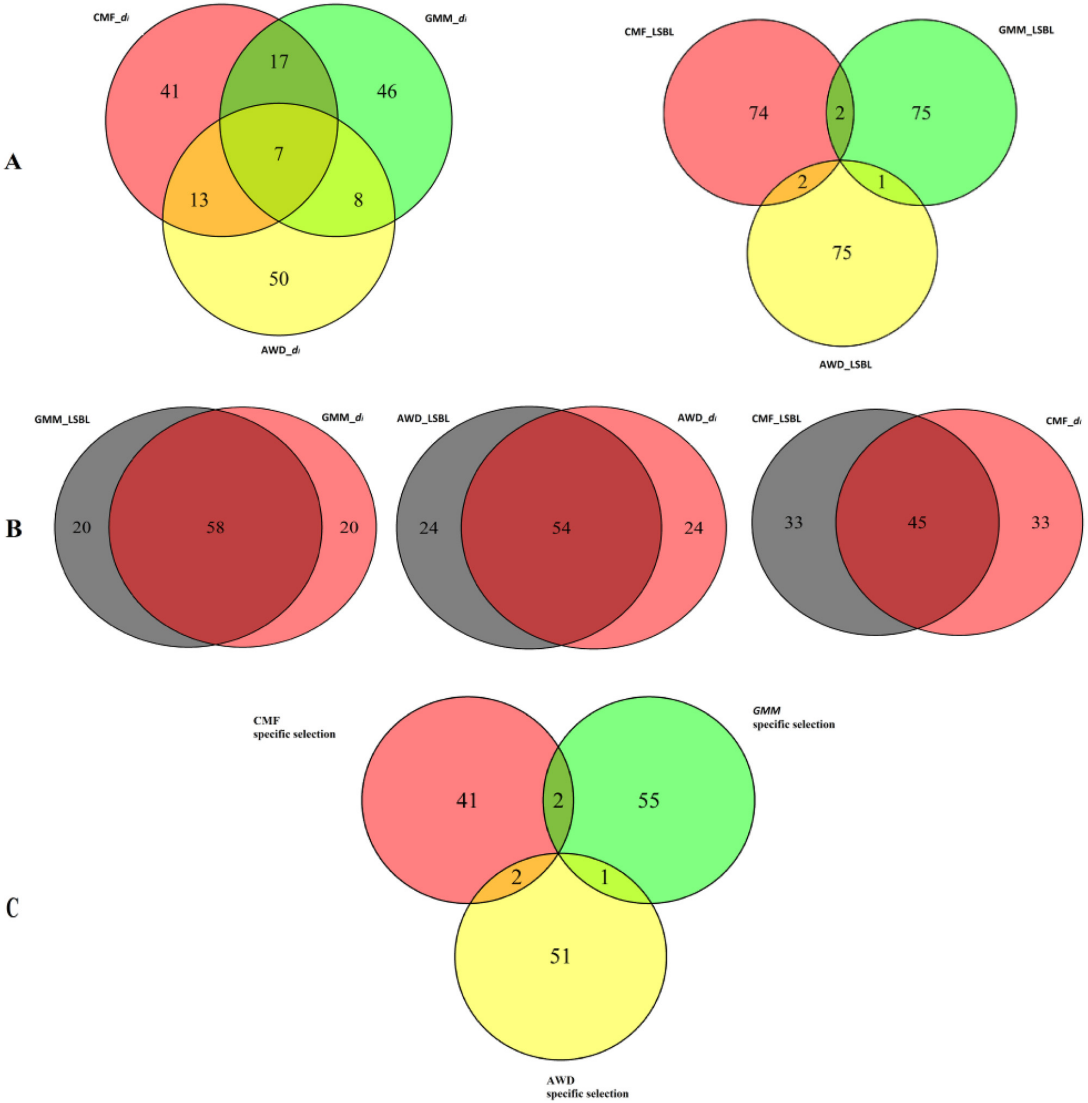
A. Former: Venn diagram of selection windows from *d*_*i*_ approach in three breeds, Latter: Venn diagram of selection windows from LSBL approach in three breeds; B. Venn diagrams of each breed’s selection windows from LSBL and *d*_*i*_ approaches; C. Venn diagram of specific selection windows in three breeds.

For each breed, we performed two locus-specific analyses to identify candidate regions involved in selection. These two statistical methods were calculated for autosomal SNPs in 300 kb windows, with a minimum of three SNPs per window, and defining the populations by breed. In total, 46,752 SNPs were evaluated within 7734 windows, ordered from 1 to 7734, averaging 5.97 SNPs per window (SD = 1.6). We defined candidate selection regions as those that fell into the upper 99^th^ percentile of the empirical distribution. Within each breed, 78 windows were considered putative signatures of selection. S1 Fig shows the genome-wide distribution of the two analyses. In total, 259 of the windows met this criterion under both approaches in three breeds. Venn Diagrams were produced for the three breeds for LSBL and *d*_*i*_, respectively (Fig 5A). The numbers of overlapping windows for LSBL were fewer than for the *d*_*i*_ approach. This indicates that LSBL has a greater ability to detect specific selection than *d*_*i*_.

To detect breed specific selection regions for each breed, we merged the window lists generated by these two approaches to identify three subsets of 54 (AWD), 58 (GMM) and 45 (CMF) windows that showed the strongest signature of selection by displaying both high LSBL and *d*_*i*_ values (Fig 5B). Because the correlation of CMF is lower than that of AWD and GMM, the number of overlapping windows for CMF is smaller than for the other breeds. Finally, there were also five overlapping windows in the final selected windows that were selected in two breeds (Fig 5C).

**Fig 6:**
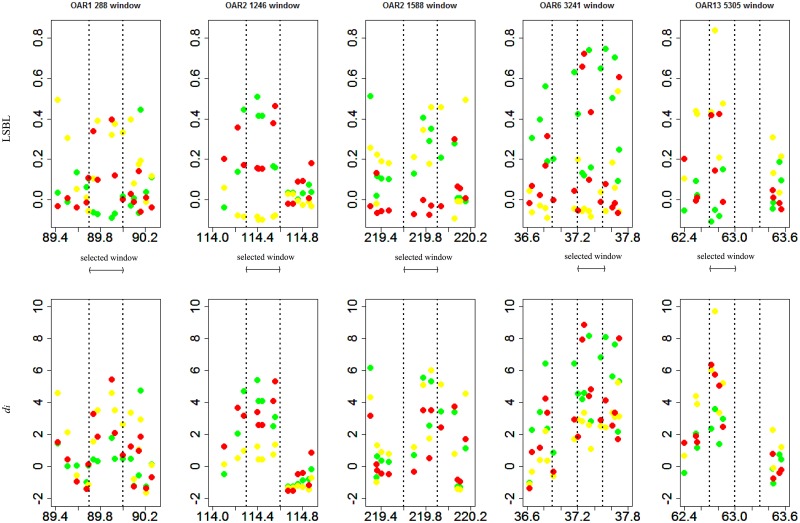
The two statistic of per-SNP of three regions with three consecutive windows, the selected widows in the middle, GMM: green dot, AWD: yellow dot, CMF: red dot.

**Fig 7:**
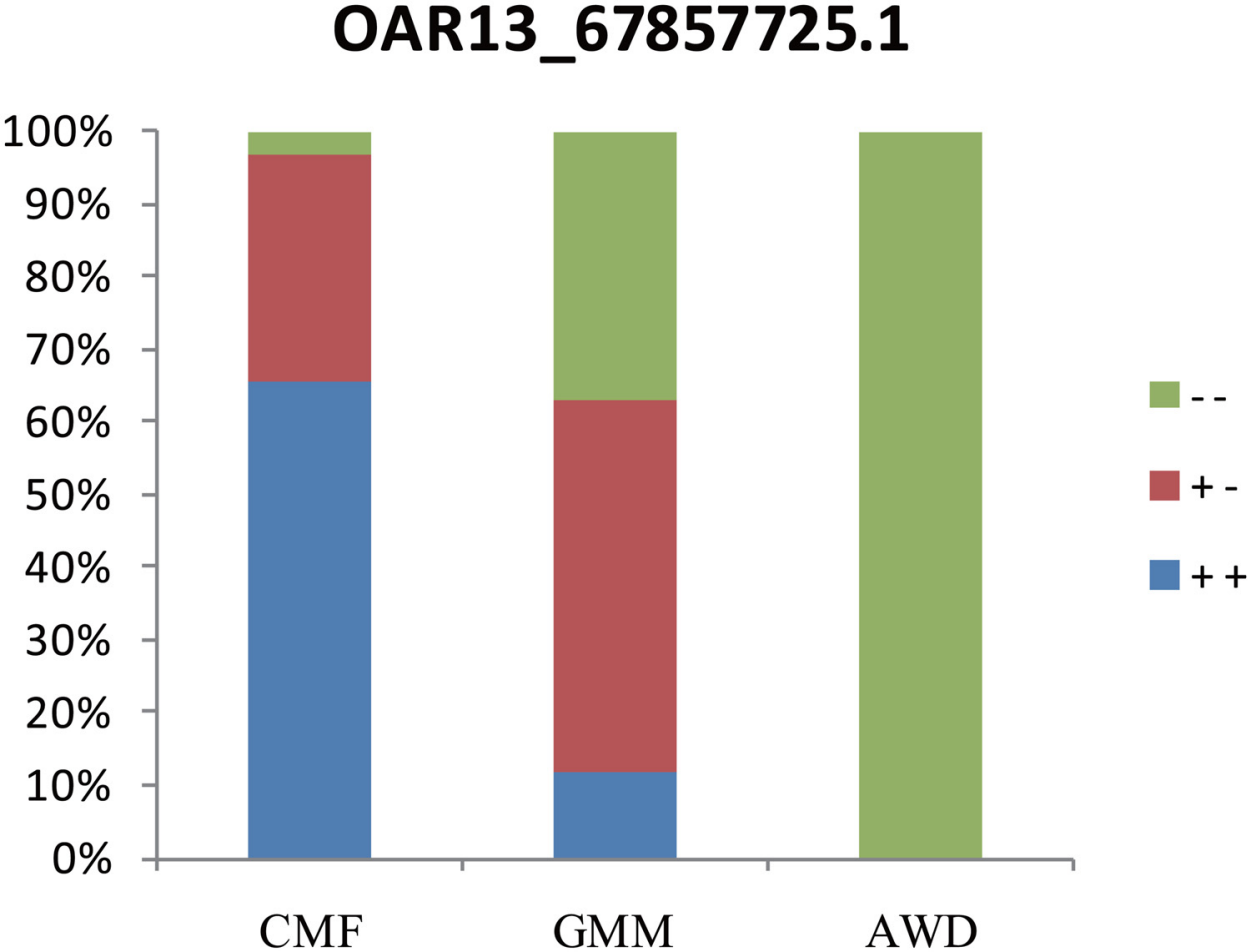
The diversity of OAR13_67857725.1 SNP in 3 sheep breeds.

Fig 6 shows LSBL and *d*_*i*_ values of-SNPs in five overlapping selection windows and in two nearby windows. The plot of LSBL values shows three clusters in each window. But these clusters are not clear in *d*_*i*_ windows. All overlapping windows include 23 SNPs. Then we investigated the diversity of these SNPs. The distribution of genotypes for each SNP in the three breeds shows a stepladder, two extreme types and one middle type (S3 Fig). Fig 7 illustrates a representative SNP (OAR13_67857725.1) in window 5305. There is clearly a large difference in genotype proportion between AWD and CMF; therefore, the overlapping selection window means the two breeds, which have overlapping selection, are different in this region and maybe one or both has undergone selection.

### Gene annotation

We used the latest sheep genome release, *Ovis_aries*_*v*3.1 (http://www.livestockgenomics.csiro.au/sheep/oar3.1.php), to identify relationships between significant selection windows and ovine genes. We removed uncharacterized genes and genes that overlapped among the three breeds. In total, 478 non-overlapping selected genes were annotated and 164, 201and 113 were selected in GMM, AWD and CMF breed, respectively (Table 2). Because of selective sweep or hitchiking effort, the effect of a strongly selected allele at one locus on the frequencies of neutral alleles at a linked locus, fewer genes were in fact selected[12]. We performed a further screen for each selection window. We selected genes located in or near a peak value SNP in each selection window. At last, we got 46, 51 and 32 candidate genes for GMM, AWD and CMF breed, respectively (Table 2, S1, S2 and S3 Tables). We did not screen overlapping windows.

**Table 2:** The annotation details in specific selected and overlapping selected region.

	Breed	No. of selected windows	No. of genes in windows	No. of genes within or near Peak SNP
overlapping selected				
	GMM & AWD	2	9	
	GMM& CMF	2	3	
	AWD& CMF	1	5	
specific selected				
	GMM	55	164	46
	AWD	51	201	51
	CMF	41	113	32

### Specific selection genes in each breed

Here we identified many selection genes for each breed. We focused on production, meat, reproduction and health traits because these are highly valued traits in mutton sheep production. We identified candidate genes are for enrichment of these main traits. We list below some genes previously identified to be important in each breed for various traits (Table 3).

**Table 3:** The information of main candidate gene of three breeds.

Breed	Window	Chr	Region	LSBL	*d*_*i*_	candidate gene
GMM						
	746	1	234.6–234.9	0.33	3.20	IGSF10
	764	1	240–240.3	0.34	4.22	PLSCR2
	1574	2	213.6–213.9	0.43	4.03	FAM113B
	1881	3	61.8–62.1	0.38	3.31	EDAR
	1984	3	93.9–94.2	0.42	4.61	EXOC6B
	2150	3	145.2–145.5	0.37	3.78	PDZRN4
	2212	3	165–165.3	0.35	3.29	NTN4
	2366	3	213–213.3	0.37	3.92	MICAL3
	2999	5	66.9–67.2	0.36	4.24	CCNB2
	3454	6	103.2–103.5	0.42	3.93	STK32B
	4579	10	55.2–55.5	0.34	3.31	EIF3F
	4794	11	39.9–40.2	0.35	3.64	PSMD3,THRA,MSL1
	5342	13	74.7–75	0.32	3.10	TRHR
	5913	16	31.8–32.1	0.50	5.66	GHR
	6058	17	4.8–5.1	0.48	4.54	TMEM154
	6673	19	59.1–59.4	0.36	3.34	EEFSEC
	7019	22	15–15.3	0.30	2.85	PLCE1
	7041	22	22.2–22.5	0.33	3.28	SUFU
	7402	24	25.8–26.1	0.47	4.90	ATP2A1, APOBR
	7403	24	26.1–26.4	0.45	4.78	ALDOA
AWD						
	756	1	237.6–237.9	0.35	3.43	HMGB1
	1708	3	7.5–7.8	0.39	3.85	SPTAN1
	2001	3	99.3–99.6	0.32	2.86	IL1RL1
	2367	3	213.3–213.6	0.41	4.58	TRIOBP
	3170	6	13.8–14.1	0.32	2.97	POL
	3606	7	34.2–34.5	0.30	3.44	SPTBN5
	3749	7	78.6–78.9	0.35	2.86	SLC8A3
	4479	10	21.9–22.2	0.45	4.47	TPTE2
	4504	10	29.7–30	0.39	4.59	B3GALTL
	5270	13	51.9–52.2	0.41	3.67	TMC2
	5438	14	21.3–21.6	0.32	2.63	RPGRIP1L, FTO
	5452	14	25.5–25.8	0.34	2.95	SETD6
	5546	14	57.9–58.2	0.31	2.76	RPL7
	6360	18	29.4–29.7	0.36	3.35	CIB2
	6623	19	43.5–43.8	0.33	2.76	DNAH3
	6649	19	51.9–52.2	0.35	3.22	SCAP
	7013	22	12.3–12.6	0.33	3.75	NUDT9
CMF						
	286	1	89.2–89.4	0.32	4.30	SLC16A1
	2099	3	129.6–129.9	0.46	6.02	CRADD
	2322	3	198.6–198.9	0.24	2.55	DERA
	2858	5	22.5–22.8	0.26	2.42	SLC27A6
	3235	6	33.9–34.2	0.28	3.31	FAM190A
	3239	6	36–36.3	0.24	2.71	HERC3
	3380	6	79.5–79.8	0.27	2.73	TECRL
	3605	7	33.9–34.2	0.26	2.85	TYRO3
	3608	7	34.8–35.1	0.24	2.71	CAPN3
	4834	11	52.8–53.1	0.28	2.63	SOCS3
	5918	16	33.6–33.9	0.22	2.64	PRKAA1
	7363	24	11.7–12	0.25	2.86	SHISA9
	7407	24	27.6–27.9	0.27	3.08	PHKG1

Underlined fonts indicate candidate gene in our former GWAS study.

#### Specific selection genes in GMM breed Production traits

Two important genes *TRHR* and *APOBR* as candidate association with body mass [13, 14]. *PDS5B* showed negative covariance between average daily weight gain and backfat thickness [15]. *IGSF10* is differentially expressed in cattle with high and low residual feed intake [16]. **Meat traits:** *GHR* is a well-known gene that not only effects meat production and quality but also reproduction traits in many species [17, 18]. *STK32B* is a QTL(quantitative trait loci) for marbling score in Hanwoo [19]. *ALDOA*, which encodes a glycolytic metabolic enzyme, was expressed at around 2-fold lower levels in the longissimus muscle of Wagyu-sired fetuses at day 195 compared with Piedmontese-sired fetuses [20]. *FAM113B* is expressed in dairy cattle at least twice the level of that in beef cattle [21]. *NTN4* was down-regulated in differentiated adiposities compared with intramuscular fibroblast-like cells [22]. **Reproduction traits**: *PLSCR2* is a candidate endometrial gene in the regulation of conceptus growth and elongation [23]. *EIF3F* gene transcripts were more highly enriched in brilliant cresyl blue (BCB)+ oocytes compared with BCB− oocytes [24]. *CCNB2* was identified as significantly associated with developmental competence of bovine oocytes [25]. *PDZRN4* is associated with sperm motility of Holstein-Friesian cattle and *EEFSEC* is related to buffalo bull fertility [26, 27]. **Health traits:** *TMEM154* can reduce lentivirus susceptibility in sheep [28] and GWAS indicate this gene to be associated with susceptibility to and control of ovine lentivirus [29]. *MICAL3* is associated with immune response traits in Canadian Holstein cattle [30]. *ATP2A1* is associated with pseudomyotonia, a muscle function disorder, in cattle [31]. *PSMD3* shows significant association with the mean corpuscular volume [32]. **Other traits:** GMM is merino fine wool sheep, so wool trait was also selected when in process of breeding. Unsurprisingly, we found three important genes involved in wool trait. *EDAR* is associated with hair thickness in human [33]. Mutation in *Mpzl3*, a gene encoding a predicted adhesion protein, is responsible for rough coat mice with severe skin and hair abnormalities [34]. *THRA* is located at quantitative. Meanwhile, we found three genes looks association with milk traits. Such as, *EXOC6B* is a candidate gene for teat morphology and function [35]. *PLCE1* is associated with total protein weight in milk and *SUFU* is associated with the mammary system, somatic cell count and survival [36].

#### Specific selection genes in AWD breed Production traits

*FTO* is associated with BMI in human and growth rate and fat mass in pig [37–40]. *SCAP*, part of the *INSIG*-*SCAP*-*SREBP* pathway, is involved in obesity risk in Chinese children [41]. Mutations in *B3GALTL* can cause disproportionate short stature in human, and developmental delay [42]. **Reproduction traits:** *SLC8A3* is a transporter that can potentially increase the availability of L-alanine and L-histidine for gap junctional transfer in oocytes [43]. *SETD6* is involved in the transcriptional regulation of gonadotropin-releasing hormone [44]. **Health traits:** *SPTAN1* is a candidate gene for parasite resistance in livestock [45]. *CIB2* is associated with influencing interleukin levels in African Americans [46]. *HMGB1* is involved in mastitis in dairy cattle [47]. *TRIOBP* and *TMC2* can cause recessive hearing loss in human [48, 49]. *NUDT9* is a candidate gene for an inherited cataract in sheep [50]. Mutations in *SPTBN5* and *RPGRIP1L* cause retinitis pigmentosa [51, 52]. A SNP mutation in *DNAH3* is involved in recurrent airway obstruction in European horses [53]. A functional SNP in *IL1RL1* is associated with asthma in human [54]. **Other traits:** *TPTE2* may be directly or indirectly related to epithelial cells or skin development [44] and is a candidate gene associated with wool traits in Chinese Merino Sheep [55].

#### Specific selection genes in CMF breed Production traits

*TECRL* is associated with withers height in racing quarter horse [56]. *SLC27A6* is part of the peroxisome proliferator-activated receptor (PPAR) signaling pathway, which is associated with carcass conformation in cattle [57]. **Meat trait:** *FAM190A* is a QTL associated with weight after slaughter in Hanwoo cattle [58]. *CRADD* is associated with muscle compactness [59]. *PHKG1* causes high glycogen content and low meat quality in pig skeletal muscle [60]. *CAPN3* is related to meat quality traits in chickens [61]. **Reproduction traits:** *TYRO3* modulates female reproduction by influencing gonadotropin-releasing hormone [62]. *SLC16A1* plays an important role in the transport of mevalonate and ketone bodies [63] and may be involved in differences in efficiency of reproduction in cattle[64]. **Health traits:** *SOCS3* is associated with somatic cell score trait in cattle and is expressed in goat milk fat globules in response to experimental intramammary infection with *Staphylococcus aureus* [65]. **Other traits:** In milk traits, *PRKAA1* is associated with fat percentage and may have effects on fat metabolism affecting milk production traits in cattle [66]. *DERA* is a positional candidate gene for milk fat percentage in the German Holstein-Friesian population [67]. *HERC3* is associated with milk production performance in Chinese Holstein cattle [68].

### Overlapping selection regions

According to the above analysis, overlapping windows means there are differences between the two selected breeds. In Table 4, 17 selected genes in these overlapping regions are annotated.

**Table 4:** The genes in overlapping selection windows.

				LSBL			di			
Chr	window	SNP No.	Region	GMM	AWD	CMF	GMM	AWD	CMF	Selected genes
1	288	4	89.7–90.0	-0.07	0.30	0.24	0.75	3.29	3.17	LRIG2, **RPS6**
2	1246	5	114.3–114.6	0.33	-0.09	0.26	3.84	0.85	3.60	-
2	1588	4	219.6–219.9	0.29	0.30	-0.04	3.54	3.52	1.80	BCS1L, CYP27A1, **PRKAG3**, RNF25, STK36, TTLL4, **WNT10A, WNT6**, ZNF142
6	3241	6	37.2–37.5	0.37	0.00	0.31	5.20	2.55	5.14	FAM184B, **NCAPG**, **LCORL**
13	5305	4	62.7–63.0	-0.02	0.49	0.24	2.58	6.08	4.91	RALY, EIF2S2, CHMP4B

Blot fonts as candidate gene. Underlined fonts indicate values in the top first percentiles.

Firstly, two overlapping windows were detected between GMM and CMF breeds. There is no gene involved in the 1246 window. We then identified two well-known genes, NCAPG and its near neighbor LCORL within 37.2–37.5Mb on OAR6, which are reported to be involved in fetal growth, stillbirth, and carcass size in sheep and other livestock (Table 4). GWAS revealed that these two genes are associated with body weight in Australian Merino sheep[69]. Kijas et al. suggest that variation in the *NCAPG*/*LCORL* region also influences production traits in sheep [70]. In horses, GWAS indicates *LCORL*/*NCAPG* as a candidate region for withers height [71]. In cattle, *LCORL* and *NCAPG* genes are associated with feed intake and weight gain [72] and body frame size [73]. Xu et al. detected that *LCORL*/*NCAPG* have undergone positive selection in five distinct cattle breeds [74].

Secondly, there are two windows that are different between AWD and CMF breeds. One region, 89.7 to 90.0 Mb on OAR10, coincides with *LRIG2* and *RPS6* genes. *RPS6* is a candidate gene in a QTL region affecting growth and reproduction traits in swine [75]. The other region, from 62.7 to 63.0 Mb, on OAR13 included three genes, *RALY*, *EIF2S2* and *CHMP4B* (Table 4). Another nearby gene, *ASIP*, regulates pigmentation in mice, while duplication of ASIP in sheep controls a series of alleles for black and white coat color [76]. The *ASIP* region is one of four known melanoma-susceptibility regions and includes the four genes (*RALY*, *EIF2S2*, *CHMP4B* and *ASIP*) [77]. In Kijas et al. research, a SNP s51670.1 has peak value of global F_ST_ in similar region, ASIP as candidate gene, on OAR13 [78]; here this SNP also has peak value of LSBL and *d*_*i*_ in 5305 windows (Fig 7). In a recent GWAS analysis ASIP was associated with white versus non-white coat-color variation in sheep [79].

Thirdly, only one region was different between AWD and GMM, at 219.6–219.9 Mb on OAR4. There are nine genes involved (Table 4), three of which have been are reported. The most important gene is *PRKAG3* (protein kinase, AMP-activated, gamma 3 noncatalytic subunit), which increases fatty acid oxidation and glucose uptake to satisfy muscle energy demands [80] and is a candidate gene associated with meat quality and production traits in pig [81] and cattle [82]. A mutation in *PRKAG3* is associated with excess glycogen content in pig skeletal muscle [83]. Recently GWAS analysis indicated that *PRKAG3* affected meat pH and color in crossbred commercial pig lines [84]. The other two genes are *WNT10A* and *WNT6*, which are strongly co-expressed in human SW480 cells [85]. Wnt6 is an early negative regulator of limb chondrogenesis and ectoderm development in the chicken embryo [86]. Interestingly, Christodoulides et al. identified a proband with early onset obesity that is heterozygous for a *WNT10* C256Y mutation, which blocks adipogenesis [87].

## Discussion

Three breeds of sheep were investigated in this study; CMF comes from China, GMM originates from Germany and AWD was originally developed in South Africa. The F_ST_ results showed significant genetic divergence between GMM and AWD (F_ST_ = 0.19) and medium divergence between CMF and GMM (F_ST_ = 0.13) or AWD (F_ST_ = 0.14). This is consistent with domestic sheep being first domesticated in Asia, the Fertile Crescent, and then dispersing to Europe and Africa [88]. The PCA and neighbor-joining tree clearly separate these three population samples from each other.

In the present study, we used two locus specific analysis approaches to detect candidate regions targeted by selection. Both of them calculated for each breed based on pairwise F_ST_. From the previously describe, the *d*_*i*_ approach measures the standardized locus-specific deviation in levels of population structure [4]. However, the LSBL approach geometrically isolates allele frequency change [3].

First we compared the values of these two statistical approaches. The two methods had a high correlation, especially in the selected regions. For example, the highest correlation (r>0.9) occurred in the region of the top 1–5000 SNPs by LSBLs in all three breeds. Furthermore, the result shows the high correlation of LSBL and *d*_*i*_ in AWD and GMM, while lower in CMF. It might be relevant with the evolution process of these three breeds. AWD and GMM are notable commercial breeds in the world, which developed through strict selection pressure. However, CMF is local breed which mainly selected for body weight and conformation in recent years [5].

We then calculated the mean value respectively of the two approaches for autosomal SNPs in 300 kb windows for each breed. Interestingly, LSBL had a greater ability to detect specific selection than *d*_*i*_. We merged the window lists generated by these two approaches to identify breed specific selection regions. In total, 142 windows showed the strongest signature of selection, five of which overlapped. This means that the two breeds are different in these regions and one or both may have undergone selection.

We have defined candidate genes in selection windows located at or near a peak value SNPs. Some genes were identified in earlier sheep selection studies, such as *NF1* and *ASIP* [78], *RNF180* and *GHR* [89]. *GHR*, identified in the GMM breed, is an important growth-related gene that, not only affects meat production and quality, but also reproduction traits [17, 18]. Two genes were detected in sheep by GWAS, such as *TPTE2* [55], *TMEM154* [29]. In our previous study four genes, *POL*, *RPL7*, *MSL1* and *SHISA9*, are associated with growth and meat production traits [5]. We notice that there are only a littler common results in these two studies, although using the same data. Because the sample sizes were too small, we combined three population data as a whole object in our GWA study. But herein, we respectively detected the specific selection for each breed.

Therefore, our study provides additional information for interpreting selection in different domestic sheep breeds. Production, meat, reproduction and health traits of sheep were investigated because these are highly valued traits in mutton sheep production. So the candidate genes enrich for these main traits. For production traits, there are two genes, *APOBR* and *FTO*, are associated with BMI [14, 37]. For reproduction traits, we found no major genes controlling reproduction prolificacy, such as *GDF9* and *BMPR1B*; however, we found some genes which can influence development of the oocyte and sperm. For example, *EIF3F*, *CCNB2* and *SLC8A3* affect oocyte development [24, 25, 43] and *PDZRN4* and *EEFSEC* affect sperm [26, 27]. For meat traits, *ALDOA*, *STK32B* and *FAM190A* are related to marbling in cattle[19, 20, 58]. For wool traits, EDAR was selected in the GMM breed and is associated with hair thickness [33]. AWD has a characteristic of molting, and *TPTE2* is related to epithelial cells or skin development [44]. For health traits, we noticed that association of candidate genes related to disease resistance traits is more common in Chinese compared with Mongolian commercial mutton sheep. This shows that the artificial selection of Mongolian sheep has not received sufficient attention. An important gene was found, *TMEM154*, which can control and reduce lentivirus susceptibility in sheep [28, 29]. Currently, there is no vaccine to prevent ovine lentivirus infection and no cost-effective treatment for infected animals. This gene should therefore be used in breeding projects. In the AWD breed, we found a lot of genes associated with disease (except for immune related genes). These included sensory disorders and respiratory system diseases. Interestingly, some genes related to milk traits were selected in GMM and CMF breeds, both of which are from the Northern hemisphere, but not in AWD.

It is worth mentioning that the early growth speed of Chinese Mongolian sheep is too slow compared with commercial breeds. This is because the Chinese Mongolian sheep is a fat-tailed sheep and deposition of tail fat reduces early growth speed. We therefore focused on the pathways and genes associated with fat formation. Interestingly, five such genes (*SOCS2*, *SOCS3*, *PPP1CC*, *PHKG1* and *PRKAA1*) are in the insulin signaling pathway. *SOCS2* and *SOCS3* (suppressor of cytokine signaling 2 and 3), regulate insulin signaling in different tissues by impacting on the insulin receptor and insulin receptor substrates [90]. *PPP1CC*, also known as *PPP1G*, is a subunit of protein phosphatase 1. It is a glycogen-associated phosphatase responsible for dephosphorylation and subsequent inactivation of glycogen synthase and is universal in skeletal muscle [91]. *PHKG1*, causes high glycogen content and low meat quality in pig skeletal muscle [60]. PRKAA1/2 acts as an energy sensor, sensing an increased AMP/ATP ratio, and is known to regulate substrates that mediate metabolic activity, such as phosphorylation of acetyl coA carboxylase (ACACA, also known as ACC) [92]. Furthermore, studies have shown that *PDGF* promotes proliferation and inhibits differentiation of preadipocytes [93, 94]. Real-time quantitative PCR indicates that *PDGFD* is expressed at a higher level in adipose tissue than in normal human tissues, except the thyroid [95]. Insulin also stimulates cell growth and differentiation, and promotes the storage of substrates in fat, liver and muscle by stimulating lipogenesis, glycogen and protein synthesis, and inhibiting lipolysis, glycogenolysis and protein breakdown [96]. We therefore suggest that these genes affect fat-tail formation but this requires further study.

In this study, we also found some different selection regions between breeds; however, we were unable to determine in which breed the candidate gene was selected. For instance, CMF has a black head and legs, while the AWD are white. It appears as though *ASIP*, a key gene of pigmentation, may provide evidence for selection in CMF. According to the same principle, the *LCORL*/*NCAPG* region was selected in GMM, which grows faster and has a bigger carcass than CMF. Of course, not all genes can be judged, such like *PRKAG3* affecting meat pH and color, because the relevant data was lacking. These genes, in addition to *RPS6*, *WNT10A* and *WNT6*, require further study.

## Conclusions

In the present study, we used the two approaches, LSBL and *d*_*i*_ statistics, to detect selection regions in three different sheep breeds (populations). These approaches clearly identified selected regions in each breed, and provided many candidate genes, including some well-known genes. Overall, growth, meat and health traits are undergoing different levels of selection in these three breeds, but the choice of focus differs for each breed according to origin, local preferences and environment.

## Supporting Information

S1 Fig Neighbor-Joining (NJ) phylogeny for 322 sheep. (TIF)

S2 Fig Genomic distribution of LSBL and di in 3 sheep breeds. (TIF)

S3 Fig The diversity of 23 SNPs of 3 sheep breeds. (TIF)

S1 Table The main candidate genes of specific selections in GMM. (DOCX)

S2 Table The main candidate genes of specific selections in AWD. (DOCX)

S3 Table The main candidate genes of specific selections in CMF. (DOCX)
