# Supplementary figures and images for: Genome-Wide Specific Selection in Three Domestic Sheep Breeds
Source: PLoS One. 2015 Jun 17;10(6):e0128688. doi: 10.1371/journal.pone.0128688 (PMC4471085; doi:10.1371/journal.pone.0128688)

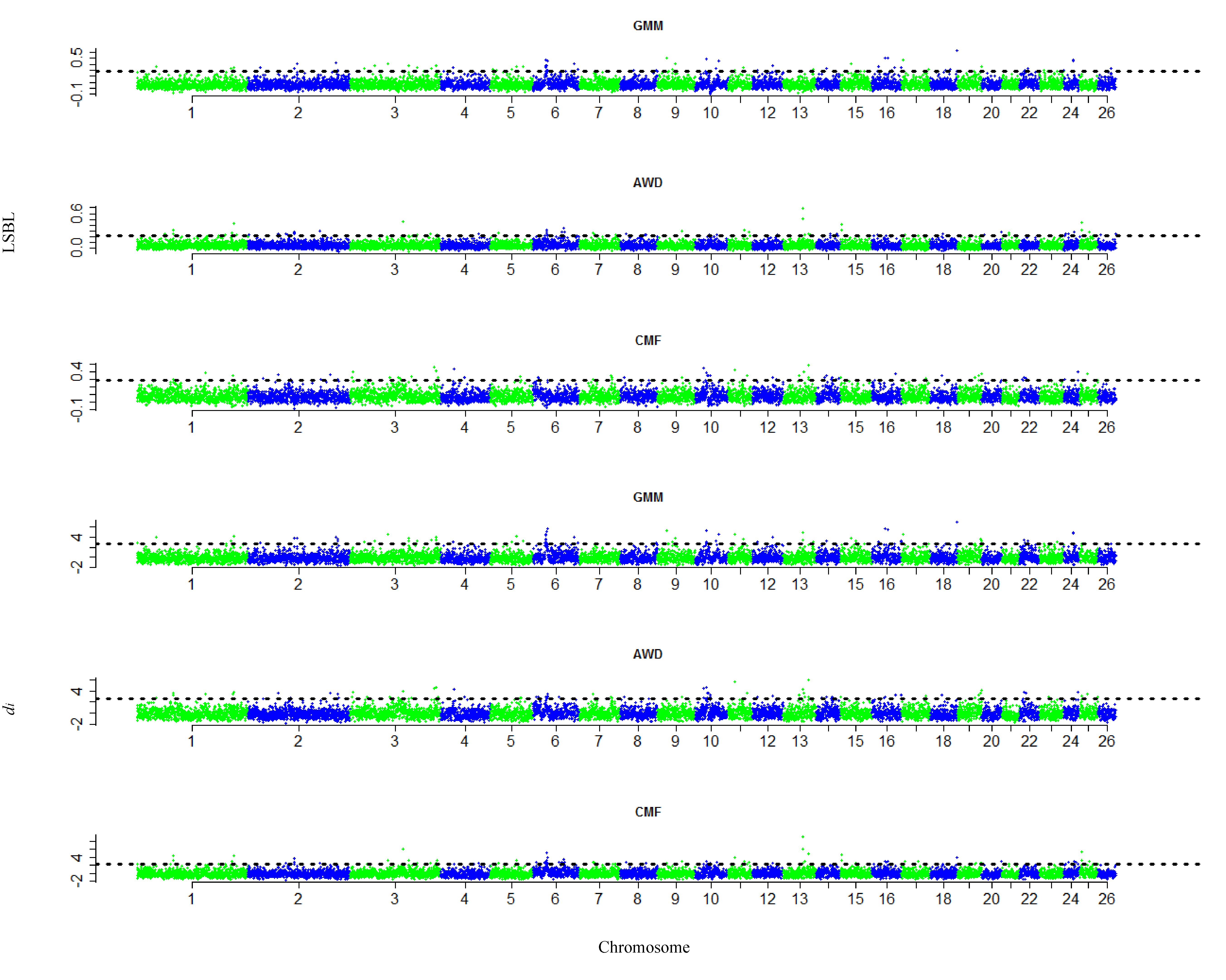

Supplement: S2 Fig — (TIF) [file pone.0128688.s002.tif]
