# Supplementary material for: Genome-Wide Specific Selection in Three Domestic Sheep Breeds
Source: PLoS One. 2015 Jun 17;10(6):e0128688. doi: 10.1371/journal.pone.0128688 (PMC4471085; doi:10.1371/journal.pone.0128688)
Supplement: S1 Table — (DOCX) [file pone.0128688.s004.docx]

S1 Table. The main candidate genes of specific selections in GMM

| Window | Chr | Region | LSBL | *d_i_* | Candidate gene |
| --- | --- | --- | --- | --- | --- |
| 746 | 1 | 234.6-234.9 | 0.33 | 3.20 | IGSF10 |
| 764 | 1 | 240-240.3 | 0.34 | 4.22 | PLSCR2 |
| 1574 | 2 | 213.6-213.9 | 0.43 | 4.03 | FAM113B |
| 1586 | 2 | 219-219.3 | 0.29 | 3.19 | TMBIM1 |
| 1588 | 2 | 219.6-219.9 | 0.29 | 3.54 | CYP27A1 |
| 1798 | 3 | 35.4-35.7 | 0.33 | 3.10 | WDR43,TRMT61B |
| 1881 | 3 | 61.8-62.1 | 0.38 | 3.31 | EDAR |
| 1984 | 3 | 93.9-94.2 | 0.42 | 4.61 | EXOC6B |
| 2150 | 3 | 145.2-145.5 | 0.37 | 3.78 | PDZRN4 |
| 2212 | 3 | 165-165.3 | 0.35 | 3.29 | NTN4 |
| 2323 | 3 | 198.9-199.2 | 0.32 | 3.36 | EPS8 |
| 2366 | 3 | 213-213.3 | 0.37 | 3.92 | ATP6V1E1,MICAL3 |
| 2814 | 5 | 6.6-6.9 | 0.31 | 2.76 | OR10H1 |
| 2999 | 5 | 66.9-67.2 | 0.36 | 4.24 | CCNB2 |
| 3055 | 5 | 84.3-84.6 | 0.36 | 3.23 | CCNH |
| 3228 | 6 | 31.5-31.8 | 0.28 | 3.53 | GRID2 |
| 3454 | 6 | 103.2-103.5 | 0.42 | 3.93 | STK32B |
| 3487 | 6 | 113.4-113.7 | 0.32 | 2.78 | SORCS2 |
| 4254 | 9 | 44.1-44.4 | 0.41 | 3.72 | ARFGEF1 |
| 4500 | 10 | 28.5-28.8 | 0.49 | 5.36 | PDS5B |
| 4579 | 10 | 55.2-55.5 | 0.34 | 3.31 | EIF3F |
| 4794 | 11 | 39.9-40.2 | 0.35 | 3.64 | PSMD3, THRA,MSL1 |
| 5025 | 12 | 53.4-53.7 | 0.38 | 3.95 | RABGAP1L |
| 5342 | 13 | 74.7-75 | 0.32 | 3.10 | TRHR,SLC13A3 |
| 5641 | 15 | 28.2-28.5 | 0.40 | 3.87 | TMPRSS4, MPZL3 |
| 5858 | 16 | 15-15.3 | 0.37 | 3.08 | RNF180 |
| 5913 | 16 | 31.8-32.1 | 0.50 | 5.66 | GHR |
| 6042 | 16 | 71.4-71.7 | 0.34 | 3.06 | LPCAT1,CLPTM1L |
| 6058 | 17 | 4.8-5.1 | 0.48 | 4.54 | TMEM154 |
| 6255 | 17 | 66.9-67.2 | 0.31 | 2.77 | TTC28 |
| 6478 | 18 | 66.3-66.6 | 0.62 | 6.97 | CDC42BPB,EXOC3L4 |
| 6673 | 19 | 59.1-59.4 | 0.36 | 3.34 | EEFSEC |
| 7019 | 22 | 15-15.3 | 0.30 | 2.85 | PLCE1 |
| 7041 | 22 | 22.2-22.5 | 0.33 | 3.28 | SUFU |
| 7270 | 23 | 44.4-44.7 | 0.29 | 3.06 | SETBP1 |
| 7402 | 24 | 25.8-26.1 | 0.47 | 4.90 | ATP2A1, APOBR |
| 7403 | 24 | 26.1-26.4 | 0.45 | 4.78 | ALDOA |
