# Supplementary material for: Genome-Wide Specific Selection in Three Domestic Sheep Breeds
Source: PLoS One. 2015 Jun 17;10(6):e0128688. doi: 10.1371/journal.pone.0128688 (PMC4471085; doi:10.1371/journal.pone.0128688)
Supplement: S2 Table — (DOCX) [file pone.0128688.s005.docx]

S2 Table. The main candidate genes of specific selections in AWD

| Window | Chr | Region | LSBL | *d_i_* | Candidate gene |
| --- | --- | --- | --- | --- | --- |
| 539 | 1 | 168.6-168.9 | 0.39 | 3.47 | CBLB |
| 756 | 1 | 237.6-237.9 | 0.35 | 3.43 | HMGB1 |
| 1118 | 2 | 74.1-74.4 | 0.37 | 3.56 | KDM4C |
| 1211 | 2 | 102.6-102.9 | 0.30 | 2.70 | FBXO3, |
| 1525 | 2 | 198.6-198.9 | 0.36 | 3.56 | MOBKL1 |
| 1698 | 3 | 4.5-4.8 | 0.32 | 3.01 | NTNG2 |
| 1708 | 3 | 7.5-7.8 | 0.39 | 3.85 | WDR34,SPTAN1 |
| 2001 | 3 | 99.3-99.6 | 0.32 | 2.86 | IL1RL1 |
| 2348 | 3 | 207.6-207.9 | 0.47 | 4.40 | LAG3,MLF2 |
| 2367 | 3 | 213.3-213.6 | 0.41 | 4.58 | TRIOBP |
| 2381 | 3 | 217.5-217.8 | 0.33 | 2.90 | ARFGAP3 |
| 2508 | 4 | 32.1-32.4 | 0.44 | 4.24 | PGY2 |
| 2585 | 4 | 56.4-56.7 | 0.33 | 3.00 | DOCK4 |
| 3037 | 5 | 78.3-78.6 | 0.29 | 2.69 | RASGRF2 |
| 3170 | 6 | 13.8-14.1 | 0.32 | 2.97 | POL |
| 3606 | 7 | 34.2-34.5 | 0.30 | 3.44 | SPTBN5 |
| 3749 | 7 | 78.6-78.9 | 0.35 | 2.86 | SLC8A3 |
| 3750 | 7 | 78.9-79.2 | 0.32 | 2.76 | SYNJ2BP,MED6 |
| 4479 | 10 | 21.9-22.2 | 0.45 | 4.47 | TPTE2 |
| 4504 | 10 | 29.7-30 | 0.39 | 4.59 | B3GALTL |
| 4511 | 10 | 31.8-32.1 | 0.35 | 3.60 | FLT1 |
| 4512 | 10 | 32.1-32.4 | 0.36 | 3.58 | PAN3,FLT3 |
| 4725 | 11 | 18.3-18.6 | 0.42 | 5.60 | NF1 |
| 4825 | 11 | 50.1-50.4 | 0.35 | 3.67 | MAFG,SIRT7, GCGR |
| 5022 | 12 | 51.6-51.9 | 0.32 | 3.03 | CSF2RA |
| 5233 | 13 | 39-39.3 | 0.35 | 3.23 | RALGAPA2 |
| 5270 | 13 | 51.9-52.2 | 0.41 | 3.67 | TMC2 |
| 5415 | 14 | 13.5-13.8 | 0.29 | 2.72 | CBFA2T3,ACSF3 |
| 5438 | 14 | 21.3-21.6 | 0.32 | 2.63 | RPGRIP1L, FTO |
| 5452 | 14 | 25.5-25.8 | 0.34 | 2.95 | SETD6 |
| 5495 | 14 | 39.6-39.9 | 0.32 | 2.89 | CCNE1,RMP |
| 5546 | 14 | 57.9-58.2 | 0.31 | 2.76 | RPL7 |
| 5564 | 15 | 2.4-2.7 | 0.33 | 2.85 | MRPL32 |
| 6360 | 18 | 29.4-29.7 | 0.36 | 3.35 | CIB2 |
| 6623 | 19 | 43.5-43.8 | 0.33 | 2.76 | DNAH3 |
| 6649 | 19 | 51.9-52.2 | 0.35 | 3.22 | SCAP |
| 6676 | 19 | 60-60.3 | 0.38 | 4.15 | CHCHD6 |
| 7013 | 22 | 12.3-12.6 | 0.33 | 3.75 | NUDT9 |
| 7023 | 22 | 16.5-16.8 | 0.31 | 3.56 | CCNJ |
| 7434 | 24 | 36.3-36.6 | 0.40 | 3.85 | TRIM4,CYP3A28 |
| 7509 | 25 | 19.2-19.5 | 0.38 | 3.39 | REEP3 |
