# Supplementary material for: Genome-Wide Specific Selection in Three Domestic Sheep Breeds
Source: PLoS One. 2015 Jun 17;10(6):e0128688. doi: 10.1371/journal.pone.0128688 (PMC4471085; doi:10.1371/journal.pone.0128688)
Supplement: S3 Table — (DOCX) [file pone.0128688.s006.docx]

S3 Table. The main candidate genes of specific selections in CMF

| Window | Chr | Region | LSBL | *d_i_* | Candidate gene |
| --- | --- | --- | --- | --- | --- |
| 286 | 1 | 89.2-89.4 | 0.32 | 4.30 | SLC16A1 |
| 1245 | 2 | 114-114.3 | 0.24 | 2.69 | EEF1A1 |
| 2099 | 3 | 129.6-129.9 | 0.46 | 6.02 | SOCS2, CRADD |
| 2322 | 3 | 198.6-198.9 | 0.24 | 2.55 | DERA |
| 2371 | 3 | 214.5-214.8 | 0.23 | 3.23 | CBX7, PDGFB |
| 2698 | 4 | 90.6-90.9 | 0.24 | 2.41 | GRM8 |
| 2858 | 5 | 22.5-22.8 | 0.26 | 2.42 | SLC27A6 |
| 3235 | 6 | 33.9-34.2 | 0.28 | 3.31 | FAM190A |
| 3239 | 6 | 36-36.3 | 0.24 | 2.71 | HERC3, NAP1L5 |
| 3380 | 6 | 79.5-79.8 | 0.27 | 2.73 | TECRL |
| 3605 | 7 | 33.9-34.2 | 0.26 | 2.85 | RPAP1,TYRO3 |
| 3608 | 7 | 34.8-35.1 | 0.24 | 2.71 | CAPN3,ZFP106 |
| 3866 | 8 | 13.8-14.1 | 0.24 | 2.64 | NKAIN2 |
| 4021 | 8 | 62.7-63 | 0.22 | 2.46 | TNFAIP3 |
| 4802 | 11 | 42.6-42.9 | 0.30 | 2.91 | DHX8,ETV4 |
| 4834 | 11 | 52.8-53.1 | 0.28 | 2.63 | SOCS3 |
| 5263 | 13 | 48.9-49.2 | 0.70 | 9.13 | PPP1CC |
| 5275 | 13 | 53.4-53.7 | 0.22 | 2.95 | EEF1A2,KCNQ2 |
| 5568 | 15 | 3.6-3.9 | 0.30 | 3.20 | PDGFD |
| 5918 | 16 | 33.6-33.9 | 0.22 | 2.64 | PRKAA1 |
| 6832 | 20 | 49.2-49.5 | 0.27 | 3.03 | BPHL,RIPK1 |
| 7363 | 24 | 11.7-12 | 0.25 | 2.86 | SHISA9 |
| 7407 | 24 | 27.6-27.9 | 0.27 | 3.08 | PHKG1 |
| 7527 | 25 | 24.6-24.9 | 0.28 | 2.92 | TET1 |
